# Working together to achieve greater quality and safety in healthcare; interweaving Quality Improvement, Lean Six Sigma and Human Factors Ergonomics: a viewpoint paper

**DOI:** 10.1108/IJHCQA-06-2025-0077

**Published:** 2026-01-09

**Authors:** Marie Elizabeth Ward, John Fitzsimons, Seán Paul Teeling, Nick McDonald, Una Geary, Margaret Codd, Mary Browne, Angela O’Dea

**Affiliations:** Quality and Safety Improvement Directorate, St James’s Hospital, Dublin 8, Ireland; Centre for Innovative Human Systems, School of Psychology, Trinity College, Dublin, Ireland; Children’s Health Ireland at Temple Street, Dublin, Ireland; School of Nursing, Midwifery and Health Systems, University College Dublin, Dublin, Ireland; Centre for Innovative Human Systems, Trinity College Dublin, Dublin, Ireland; Health Service Executive, Dublin, Ireland; The Irish Center of Applied Patient Safety and Simulation, School of Medicine, University of Galway, Galway, Ireland

**Keywords:** Lean Six Sigma, Quality Improvement, Human Factors Ergonomics

## Abstract

**Purpose:**

Many efforts to improve patient safety across the healthcare system over the last 20 years have not met their desired objectives. Transformational and courageous leadership is needed to encourage thinking at the macro, meso and micro system levels to identify what are the best approaches to change at different system levels that will ultimately enable improvements in patient safety. In this viewpoint we explore Quality Improvement (QI), Lean Six Sigma (LSS) and Human Factors Ergonomics (HFE), as approaches to change and improvement.

**Design/methodology/approach:**

As authors we are experts in QI, LSS and HFE and this paper has arisen out of much dialogue between us aimed at developing a shared understanding and appreciation of QI, LSS and HFE and how these approaches could work together towards the goal of improved quality of care and patient safety. We draw on the literature and case studies that we have authored using these three approaches.

**Findings:**

The findings are presented along a number of key areas including: defining the approaches and understanding their theoretical underpinnings; outlining what type of changes are suited to each approach including giving some case study examples; outlining how each approach tackles key issues in change and improvement including understanding context, developing, implementing and evaluating change.

**Practical implications:**

We believe that QI, LSS and HFE approaches to change are complementary and synergistic and that they do not contradict each other. Through prioritising the well-being of both patients and staff, and fostering cultures that promote collaboration, innovation and resilience, they can enable healthcare organisations to more effectively achieve their goals of improved safety, efficiency and human well-being. In this viewpoint we propose that there is room for the three approaches to be adopted in healthcare for different purposes and at different system levels depending on existing capability and capacity.

**Originality/value:**

Sharing understanding of QI, LSS and HFE and what each approach can offer, respecting the complementarity between them and the possibility of achieving more by working together and interweaving these approaches, can help us all to reap greater benefits for patient safety and care quality.

## Introduction

Many efforts to improve patient safety across the healthcare system over the last 20 years have not met their desired objectives (Wears and Sutcliffe, 2019). New ways of thinking and approaches are needed to tackle the problem. The World Health Organization (WHO) has called for healthcare to embrace the science of Human Factors Ergonomics (HFE) to strengthen the resilience of healthcare organisations and clinical practices. It also calls for transformational leaders to identify, and address issues in the design, organisation and delivery of services that will have the biggest impact on the safety and resilience of services (WHO, 2021). Out of 108 countries surveyed in a recent analysis no country has taken up this challenge at a healthcare system level (WHO Global Patient Safety Report, 2024). There still exists confusion over what HFE is and how it relates to other approaches and frameworks for change and improvement. In this viewpoint, we build on the work of Hignett *et al.* (2015) who explored strategies for the integration of HFE and Quality Improvement (QI) and that of Scoville and Little (2014) who compared Lean and QI. We bring this work forward by exploring the complementarity between HFE and both QI and Lean Six Sigma (LSS) in improving healthcare quality and patient safety. While many change frameworks are applied in healthcare, we chose to add LSS to this discussion due to the rising popularity of its use in healthcare over the last 10 years. This viewpoint is written with transformational leaders in mind, many of whom may already be familiar with either QI or LSS, to support them in leveraging the best of these three different approaches towards the goal of strengthening healthcare system resilience.

## How do we define QI, LSS and HFE?

QI is the combined and continuous efforts of everyone (all who influence the patient’s journey) to make changes that positively affect the domains of quality (safe, effective, patient-centred, timely, efficient and equitable) in ways that lead to better patient outcomes, better system performance and better professional development (adapted from Batalden and Davidoff, 2007; IOM, 2001). Lean is a QI methodology that emphasises improving flow and eliminating non-value add (NVA) activities – defined as any steps, processes, or actions that do not contribute value as perceived by the customer. A person-centred approach to Lean extends this focus by aligning improvement efforts with individuals’ experiences and needs, ensuring that changes are both effective and meaningful. Six sigma is a data-driven process improvement methodology. A hybrid of Lean and Six Sigma as LSS appears in the healthcare literature from 2010 onwards (Teeling *et al*., 2021). HFE is the scientific discipline concerned with the understanding of interactions among humans and other elements of a system, and the profession that applies theory, principles, data and methods to design in order to optimise human well-being and overall system performance (IEA, 2000).

## What are the theoretical underpinnings of QI, LSS and HFE?

QI draws on management theory and in particular Deming’s system of profound knowledge (SoPK) which includes systems thinking, measurement to understand variation, the design of experiments to test change, along with individual and social psychology (Deming, 2018). The initial focus of QI was on specific domains of quality (safety, timeliness), delivering improvement quickly through projects and campaigns. Over time there has been a shift to try and address all the domains holistically and highlighting neglected domains such as equity. Recently there has been a move towards “Quality Management Systems”, paying attention to sustainability and spread of improvement through planning or design for quality as well as quality control once improvements have been made (“the Juran trilogy”). There has also been an appreciation of the need to include all the people involved in healthcare (the “everyone” in QI) when decisions are being made about what should be improved and how it might be done (co-production) (Batalden *et al.*, 2016).

LSS draws on both the fundamentals of QI and management theory and the concept of process flow. Lean encompasses the SoPK in system wide, end to end, improvement. Lean is based on the philosophical underpinnings of respect for person, harmony, consensus in decision making and continuous improvement or *Kaizen*. Both Lean and Six Sigma have their origins in industry (Toyota and Motorola respectively) (Teeling *et al*., 2021).

HFE is a scientific discipline incorporating the fields of psychology, engineering, sociology, biomechanics, industrial design, physiology, anthropometry and user experience. HFE always takes a systems perspective and looks at all the elements of healthcare systems including humans, technology, tasks, equipment, processes, organisational factors and the interactions between them. Safety is a key focus, together with improvement, through deliberate design of all these components for the joint purpose of improving human well-being (staff and patients) and overall system performance. In recent years there has been a focus on developing resilient healthcare systems (RHS) that are capable of anticipating, adapting and surviving in an unstable environment (Lyng *et al.*, 2024).

## What type of changes are suited to each approach?

QI seeks to improve at least one (and ideally all) of the domains of quality (to which climate sustainability can also be added). QI is suited to addressing issues such as processes not performing as expected; patients and/or staff not happy with the existing processes; an existing process to be redesigned; new process/technology to be introduced; quality issues identified. QI can also be deployed in response to high profile problems with safety, access, cost, etc.

LSS is suited to address issues such as processes not performing; staff, suppliers, customers not happy; new process/technology to be introduced or quality, safety or risk issues identified. If the current process needs redesign, then the Define, measure, analyse, improve, control (DMAIC) approach is taken. If a new process needs to be designed then the define, measure, analyse, design, validate (DMADV) approach is taken.

HFE interventions are targeted to suit the complexity of systems and usually include complementary supporting interventions at: micro (operational group or unit), meso (middle management, technical and support functions) and macro (senior management, governance) levels, as can also be the case in QI and LSS. HFE is always focused on the dual outcomes of system performance and human wellbeing. Other areas of focus are organisational culture including safety culture and safety and risk management. Concerns may arise from outside (e.g. research findings; best practice from other sectors, national or international reports) or inside the system.

Examples of case studies, that the authors have been involved in, are presented in Table 1. This table outlines the problem to be addressed, which of the three approaches was taken, why the approach taken was suited to this problem and what the outcome was.

**Table 1:** Case studies where QI, LSS and HFE were used to address a problem

	Problem to be addressed	Why this approach was chosen to address the problem	What was the outcome	Title of cases study and reference
QI	Rapidly improve personal protective equipment (PPE) training during the Covid-19 pandemic	Rapid learning cycles using plan-do-study-act (PDSA) cycles were used to design improvement solutions during the height of the Covid-19 crisis. Frontline staff were enabled to conduct small experiments that were safe but which yielded learning quickly to improve care (as defined by the domains)	In an East London trust in one day, the steps and decisions to procure PPE were mapped by staff, who identified a number of steps that required clarification or could be removed. A digital form was created, aligning with the new standard process, tested by one team, and then scaled throughout the organisation	Fitzsimons, J. (2021) Quality and safety in the time of Coronavirus: design better, learn faster. International Journal for Quality in Health Care, Volume 33, Issue 1, 2021, mzaa051, https://doi.org/10.1093/intqhc/mzaa051 Shah, A., Pereira, P., and Tuma, P. (2021). Quality improvement at times of crisis. *BMJ (Clinical research ed.)*, *373*, n928. https://doi.org/10.1136/bmj.n928
Person-centred Lean Six Sigma	The dermatology department faced significant challenges related to excessive outpatient waiting times. There were 3,736 patients awaiting appointments, and 1,615 patients experienced wait times exceeding 12 months. A diverse interdisciplinary team sought to address these issues while also fostering a cultural shift within the working environment to support sustainable improvements	To redesign referral, triage, and scheduling processes, a combination of Lean techniques aimed at minimising non-value-added activities and Six Sigma methodologies focused on reducing process variation was employed. To complement these technical improvements, a person-centred approach was integrated to drive the cultural transformation necessary for sustained change and engagement across the workforce	The initiative led to the establishment of a centralised triage system, enabling a more efficient allocation of appointments and improved management of urgent cases. Post-implementation data indicated a 40% reduction in the overall waiting list and a 60% decrease in the number of patients waiting over 12 months. The intervention significantly reduced waiting times across all urgency categories, with the most pronounced improvements observed in soon and urgent referrals. Furthermore, these changes served as the foundation for a subsequent design-led innovation phase, in which the team collaborated with academic and healthcare partners to facilitate disruptive, system-wide change	Implementing Person-Centred Lean Six Sigma to Transform Dermatology Waiting Lists: A Case Study from a Major Teaching Hospital in Dublin, Ireland Igoe, A., Teeling, S. P., McFeely, O., McGuirk, M., Manning, S., Kelly, V., Coetzee, H., Cunningham, Ú., Connolly, K., and Lenane, P. (2024). Sci, 6(4), 72. https://doi.org/10.3390/sci6040072
HFE	Healthcare acquired infection (HCAI) is a problem globally. A large acute hospital wanted to improve their risk management programme for the prevention and control of HCAI (PCHCAI)	PCHCAI in an acute hospital setting is a complex activity involving multiple stakeholders working across many departments of the meso system of the organisation (e.g. microbiology, occupational health, facilities management) intersecting with many different processes (e.g. screening of patients and healthcare professionals; adherence to evidence based care bundles). Any solution would need to be built on a thorough understanding of this complexity. A HFE STSA analysis was the recommended approach to understand the complexity and where to intervene to gain most leverage for improvement	In carrying out the HFE STSA of the PCHCAI system in the hospital the area of “information and knowledge” emerged as the most relevant area to gain leverage on change and improvement. A large amount of data was gathered across the hospital in relation to PCHCAI activities A deeper exploration of the governance of data, the value of different data sources and adding additional value to them through combining quality and safety data with operational data, big data analytics and machine learning, enabled the translation of all this data into knowledge for proactive risk management. This led to the risk identification, mitigation and management of one HCAI, in an acute ward with higher-than-expected incidence	A systems approach to managing the risk of healthcare acquired infection in an acute hospital setting supported by Human Factors Ergonomics, data science, data governance and AI. Ward, M. E., Geary, U., Brennan, R., Vining, R., McKenna, L., O’Connell, B., Bergin, C., Byrne, D., Creagh, D., Fogarty, M., Healy, U., McDonald, G., Ebiele, M., Crane, M., Pham, M. K., Bendechache, M., Bezbradica, M., Liang, J., Doyle, B., Guilfoyle, J., … McDonald, N. (2024). *Ergonomics*, 1–19. https://doi.org/10.1080/00140139.2024.2396527

## How is the current “as is” situation understood before the change?

QI, LSS and HFE efforts all commence with trying to understand the current contextual reality before embarking on change or improvement. Please see Table 2 for examples of how this is done. Analysis of the current “as is” may be a struggle in QI as those doing the analysis are often embedded in the existing system and may lack objectivity. In healthcare LSS is evolving to include a greater focus on person-centred culture (Teeling *et al*., 2021). There is increasing appreciation of the dual impact of people on the change and the change on people (patients and staff). LSS is also adopting a more design thinking approach to the redesign of existing processes and design of new processes.

**Table 2:** Approach to analysis and measurement

	QI	LSS	HFE
Paradigm	Pragmatic Analysis of process to understand how change will affect process	Pragmatic Analysis of process to understand how change will affect process	Scientific Analysis of system elements and system interaction using a wide range of qualitative and quantitative and experimental and observational methods and techniques
Design	Model for Improvement with PDSA cycles	Design, Measure, Analyse, Improve, Control (DMAIC)	Pre-post analysis of outcomes, experiment, action research
Approach	Usually initiated locally by frontline workers Stakeholders (staff and patients) identified and engaged to understand the “as is” situation and to create social capital through co-production Driver Diagram (Theory of Change)	Usually initiated by management and undertaken by trained staff Process Owners and Stakeholders (staff and customers) identified and engaged to understand the “as is” situation Voice of the Customer (VOC) – to understand values of all involved in the improvement process and engage them in developing a vision for the future state	Usually initiated by research collaborations with senior leaders. Often facilitated by HFE professionals (working inside or outside of the organisation) in collaboration with local quality, safety and front-line professionals and management Co-design and co-production are requisite to understand the current system (“as is”) and co-create the desired future state (“to be”)
Methods to understand what is happening before the change	Generation of a SMART (Specific, Measurable, Achievable, Relevant, and Time-Bound) Aim Statement Process mapping Observation, “common sense” insights Fishbone, 5 whys, Pareto analysis of causes Hearing the “voice of the process” through active engagement of providers and patients using methods such as Surveys, Story Telling and Liberating Structures “Steal shamelessly” and use tools from other disciplines including LSS and HFE (occasionally reduced to a principle or slogan to simplify the adoption)	Process mapping with team/stakeholders; Fishbone, 5 whys, Pareto analysis of causes, Fault Tree Analysis Gemba Walk (to visit where the process happens, observe and understand it) Risk identification and mitigation – Risk Prioritisation Matrix and Failure Modes and Effects Analysis (FMEA) Toyota Production System (TPS) – 5/6S Sort (Seiri), Set in Order (Seiton), Shine (Seiso), Standardise (Seiketsu), and Sustain (Shitsuke), Safety Concepts of Just in Time (JIT), Point of Use (POU), Right First Time (RFT) Quality Function Deployment (QFD), Critical to Quality metrics, Kano analysis Suppliers, Inputs, Process, Outputs, and Customers (SIPOC) Use of common analysis frameworks like SWOT (strengths, weaknesses, opportunities, and threats) and PESTLE (Political, Economic, Sociological, Technological, Legal and Environmental) Waste analysis, e.g. TIMWOODS/DOWNTIME Sandbox to explore ideas and Rapid Improvement Events	Assessment of Teamwork, leadership, communication, culture, wellbeing, e.g. Interviews, focus groups, surveys, ethnographic observation HFE integration into the physical built environment, the tools and technologies that humans use in their everyday work; impact of new tools and technologies or changes to the built environment How humans in the system carry out their work, e.g. Hierarchical Task Analysis (HTA) how humans in the system think about their work when they are interacting and engaging with tasks and technologies like the Electronic Patient Record, e.g. Cognitive Task Analysis (CTA); Critical Decision Method (CDM); Situational Awareness (SA) Assessment; Human Computer Interaction (HCI) assessments How current processes and systems work-as-done (WAD) and as-imagined (WAI) e.g. Process mapping and analysis; assessments of workload How humans in the system relate to each other, trust each other, work together, e.g. Analysis of social networks and roles how humans in the system share data, information and knowledge in the organisation, e.g. Mapping of information flows How the different elements of the system inter-relate and work together to create a functioning (or not) system, e.g. Socio-Technical System Analysis (STSA) How and where current processes or systems might fail, e.g. FMEA; methods for predicting and understanding how and where errors might happen, e.g. Systematic Human Error Reduction and Prediction Approach (SHERPA) Whether or not it is safe to make changes and any potential knock-on consequences (good or bad) to other parts of the system, e.g. Safety Case
Measurement of change or improvement	Assessment of process and variation over time – Run charts, statistical process control (SPC) charts Process, outcome, balancing measures used Person-centred outcome and experience measures – (PROMS and PREMS)	Use of SMART (Specific, Measurable, Achievable, Relevant, and Time-Bound) goals and objectives Critical to Quality (CTQ) to define data measurements and translate the VOC into measurable metrics Person-centred outcome and experience measures – (PROMS and PREMS)	Action research or experiment including detailed WAD vs WAI analysis Measure of impact on human wellbeing and overall system performance Ongoing measurement and pre-post analysis of outcomes including process, outcome and balancing measures Person-centred outcome and experience measures – (PROMS and PREMS)

HFE is grounded in research. It takes a methodologically rigorous approach in order to achieve a rich contextual understanding of the social and technical aspects of what is called the socio-technical system (STS). In HFE there is a strong incentive to understand how work is actually performed (“work-as-done”) as opposed to how that work is prescribed in procedures and guidelines (“work-as-imagined”). HFE systems analysis work can be resource intensive and take considerable time. Some hospitals and larger organisations are moving to having embedded HFE researchers on site (Catchpole *et al.*, 2021), which brings benefits in terms of this rich contextual understanding but also challenges in terms of retaining objectivity.

## How is the change developed in QI, LSS and HFE?

In QI an explicit theory of change is required (often represented in a driver diagram), making clear the relationships between the aim of the improvement project and the changes (drivers) to be implemented and tested. A clear aim statement, suggested changes and how to measure them are identified. The model for improvement, originally developed by associates in process control, and now promoted by the Institute for Healthcare Improvement, is used at large and small scale (Langley *et al.*, 2009). The involvement of stakeholders (patients, family members and staff) is important including in the communication plan. Co-design and co-production of change with staff and patients is encouraged in QI but approaches to co-production are still developing and challenging traditional power structures (NHS England, 2023).

In LSS, improvement comes from DMAIC and moving from the “as is” process to remove non-value add activity and unwanted variation and improve process flow. DMADV is also used for designing a product or service. Project Charters and A3 Thinking (a problem-solving, decision-making, and collaborative management tool where “A3” refers to the size of the piece of paper used to outline goals, ideas, problems and solutions) help with planning the improvement process. Other common tools for change include Sandbox to explore ideas and Rapid Improvement Events that take place over a period of a week. The involvement of stakeholders (patients, family members and staff) is key to change and is incorporated through the “Voice of Customer”. Solutions are co-designed with process owners and customers. A communication plan is developed including defining who is responsible, accountable, consulted and informed (RACI), and who the stakeholders with power and interest are.

In HFE, the change approach is determined by the outcome of the analysis of the STS elements and their interactions (Table 2) which supports a deep understanding of where to intervene in the system to gain most leverage for change (Meadows, 1999). Public and patient partnership is essential in co-designing change using human-centred design principles. Design thinking, and a co-production cycle from co-planning to co-design to co-evaluation to co-delivery are essential. HFE advocates that the impact of any proposed change in an organisation needs to be assessed including any knock-on consequences to other parts of the wider system before embarking on the change (safety case). HFE promotes an approach to managing change and safety called a “Safety Management System” which includes a safety policy and objectives; a program of safety promotion; processes for the proactive identification and management of hazards and risk; and ways of assuring safety throughout the organisation (HSSIB, 2023).

## How is the change implemented and evaluated in QI, LSS and HFE?

Implementation in QI takes place in small, experimental and incremental changes utilising plan-do-study-act (PDSA) cycles (Langley *et al.*, 2009). The change process involves tests of change and predictions about the impact of the change and measurement of the change. Advancing through small tests of change is considered important rather than trying to implement large scale change in one attempt. QI tries to engender an “improvers mind set” where every change is seen as an opportunity to learn and plan another improvement cycle. Both qualitative and quantitative measures are used to show changes. Simple graphical displays such as “safety crosses” might be used to share real-time data. Run charts and statistical process control charts are used to identify variation and measure the impact of changes. A project sponsor, who is responsible for the overall management of the project, is important in implementing the change. There can be challenges with sustaining and spreading the change unless plans for this are built into the initial project plan.

LSS implementation and evaluation involves the “Improve” and “Control” parts from DMAIC process and the “Design” and “Validate/Verify” from DMADV process. In both DMAIC and DMADV, the role of the project sponsor (executive lead) is essential as they are the person who is responsible for the overall management of the project. LSS projects often pursue the strategic objectives of the organisation. FMEAs are carried out post implementation to ensure that the change has not brought about any increased risk to the process (VHA National Centre for Patient Safety, 2021). Voice of customer (VOC) feedback is usually obtained from customers of the process or staff involved. Quality function deployment (QFD) takes place to include planning for the deployment of the new products or services developed. Post implementation “Gemba Walks” take place to evaluate the change *in situ*.

HFE implementation involves multiple interventions that tackle multiple elements of the system. HFE often involves radical change and redesign of processes, systems, culture and how people make sense of their organisation (sensemaking). HFE utilises principles from the hierarchy of controls model (NIOSH, 2024) and supports the premise of making “the right thing to do the simplest thing to do”. Evaluation is iterative and can also involve using a pre-post design in which changes to key human, organisational and system outcomes are measured following the intervention and compared to pre intervention status. In HFE, focus is also on building RHS that can change, learn, adapt and anticipate supported by effective data and information systems so the evaluation approach needs to take account of this also.

## What supports and training resources are needed for each approach?

Within QI, fidelity to principles rather than rigour of approach allows flexibility and encourages a “have a go” approach to improvement by frontline staff. Research has shown however that the successful implementation of a programme of QI projects is facilitated when the healthcare organisation engages in a capacity and capability building programme including coaching and central support. Such programmes involve four interacting components: an agreed and robust QI methodology, a skilled faculty to assist improvement teams, active involvement of leadership and management and a deep understanding that teams matter (Hibbert *et al.*, 2021).

LSS training is usually structured around white/yellow, green and black belts. LSS improvement can be carried out by staff who have training in the fundamentals (usually referred to as “White Belt” training or a one-day CPD course) and advanced training in LSS (“Green Belt” training in the form a professional certificate). LSS leaders are expected to have “Black Belt” training which involves obtaining a graduate certificate in process improvement. Most healthcare organisations who invest in LSS also set up their own LSS department to provide support for projects on the ground. Some organisations who take a whole systems approach to implementing LSS also bring their training in-house and develop their own academies.

Traditionally, people from the HFE disciplines have specialised in HFE through completing a Masters or PhD in HFE. In recent times, and given the increased recognition of what HFE can offer to improving complex systems, masters’ courses and PhD options have opened up for people from other disciplines including medical, nursing and health and social care professionals (HSCPs). Taking a HFE approach involves understanding and working with complexity at many levels of the system and designing and embedding solutions that may take longer to develop, trial and implement. HFE requires research and analysis skills to undertake a system analysis and generate an understanding of system complexity. Solutions must be pragmatic, appropriate to context, of value to stakeholders, sustainable and meet strategic objectives. Thus, HFE can be more resource intensive. Healthcare practitioners, working together with embedded HFE practitioners and researchers, can support long-term change and improvement (Catchpole *et al.*, 2021).

## How can QI, LSS and HFE weave together?

While QI, LSS and HFE each provide different frameworks for advancing healthcare systems change, they have the potential to interweave in their common purpose to facilitate the delivery of safe high-quality patient care. Each approach requires the starting point of open, human/person-centred cultures, which are key to successfully deploying any change. These types of cultures facilitate engagement, adaptability, and sustainability of change.

QI, LSS and HFE share a common belief that efforts to enhance patient safety and care quality should not become reductionist, e.g. treating improvement as merely the application of a toolkit. Instead, they are approaches to facilitate the co-creation of knowledge about current system complexity. Through prioritising the well-being of both patients and staff, and fostering cultures that promote collaboration, innovation and resilience, they can enable healthcare organisations to more effectively achieve their goals of improved safety, efficiency and human well-being.

In this viewpoint we are not suggesting that QI, LSS and HFE need to be used together for all interventions but rather that there is room for all three approaches to be adopted for different purposes and at different system levels depending on existing capability and capacity. We also believe that the three approaches are complementary and synergistic and that they do not (in principle) contradict or compete. The complexity of healthcare means there is no single approach, method or right design that will deliver on change goals, no one plan or programme that is universally true and transferrable (Barry *et al*., 2018).

HFE, as a broader framework of transdisciplinary science, can help and support specific initiatives (derived from QI or LSS or elsewhere) and build strategic capability in different ways (Dixon-Woods, 2019; Sujan *et al.*, 2021; O’Dea *et al.*, 2025). Some of those ways are mentioned here along with references to published studies that weave HFE and either QI or LSS. We could not identify any published studies that explicitly weave all three (HFE, QI and LSS) together.

1. To support change at a systems level and to synthesise improvement efforts across different specific initiatives to achieve important system wide objectives (Ward *et al.*, 2022 – HFE and LSS; Ward *et al.*, 2025 – HFE and QI).
2. To bring the rigour of research to understanding change and improvement and the barriers that need to be overcome in order to sustain and spread initiatives beyond their project phase (Wolf, 2016 – HFE and LSS);
3. To build the capability to support system wide change, spread and sustainability (Rhee *et al*., 2016; Keebler *et al*., 2023 – HFE and QI);
4. To bring together a commitment to staff wellbeing while achieving improvement goals (Vicente *et al*., 2024 – review study of HFE and LSS).
5. To understand the impact of design of the built environment on quality and safety of care (Hignett *et al.*, 2015b – HFE and LSS).
6. To more fully understand how to support RHS that are capable of anticipating, adapting and surviving in the complex environment that is healthcare (Watson *et al.*, 2022 – HFE and QI).

There are also published examples of where QI and LSS have been used together for improvement (Carman *et al*., 2014; Rollinson *et al.*, 2021). In addition, for specific HFE interventions, QI and LSS capacity and capability can be engaged and mobilised to support prioritised goals, testing and adapting to local contexts while feeding back into learning systems.

## Conclusion

QI, LSS and HFE are dynamic, complementary approaches with the potential to evolve alongside the complexities of modern healthcare systems. Sharing understanding of each approach and what it can offer, respecting the complementarity between them and the possibility of achieving more by working together and interweaving these approaches, can help us all to reap greater benefits for patient safety and care quality. Transformational and courageous leadership is needed to encourage thinking at the macro, meso and micro system levels to identify what are the best approaches to change at different system levels that will ultimately enable improvements in patient safety. Research funding is also needed to understand where and how to use different approaches to change in healthcare to maximum effect in creating whole system improvement in patient safety that will sustain over time.
